# Cross-linking mass spectrometry reveals structural insights of the glutamine synthetase from *Leishmania braziliensis*

**DOI:** 10.1590/0074-02760210209

**Published:** 2022-01-10

**Authors:** Jhenifer Yonara de Lima, Marlon Dias Mariano Santos, Mario Tyago Murakami, Paulo Costa Carvalho, Tatiana de Arruda Campos Brasil de Souza

**Affiliations:** 1 Fundação Oswaldo Cruz-Fiocruz, Instituto Carlos Chagas, Laboratório de Proteômica Estrutural e Computacional, Curitiba, PR, Brasil; 2 Centro Nacional de Pesquisa em Energia e Materiais, Laboratório Nacional de Biorrenováveis, Campinas, SP, Brasil

**Keywords:** structural proteomics, protein, glutamine synthetase, Leishmania braziliensis

## Abstract

**BACKGROUND:**

Leishmaniasis is a neglected tropical disease caused by the parasite *Leishmania braziliensis*, commonly found in Brazil and associated with cutaneous and visceral forms of this disease. Like other organisms, *L. braziliensis* has an enzyme called glutamine synthetase (LbGS) that acts on the synthesis of glutamine from glutamate. This enzyme plays an essential role in the metabolism of these parasites and can be a potential therapeutic target for treating this disease.

**OBJECTIVES:**

Investigate LbGS structure and generate structural models of the protein.

**METHODS:**

We use the method of crosslinking mass spectrometry (XLMS) and generate structural models *in silico* using I-TASSER.

**FINDINGS:**

42 XLs peptides were identified, of which 37 are explained in a monomeric model with the other five indicating LbGS dimerization and pentamers interaction region. The comparison of 3D models generated in the presence and absence of XLMS restrictions probed the benefits of modeling with XLMS highlighting the inappropriate folding due to the absence of spatial restrictions.

**MAIN CONCLUSIONS:**

In conclusion, we disclose the conservation of the active site and interface regions, but also unique features of LbGS showing the potential of XLMS to probe structural information and explore new drugs.

According to the Centers for Disease Control and Prevention (CDC), leishmaniasis comprises a group of neglected tropical diseases caused by parasites of the genus *Leishmania*. This disease presents itself in three forms: cutaneous (CL), mucous (ML), and visceral (VL). Cutaneous leishmaniasis can cause substantial morbidity, while visceral leishmaniasis can be fatal.^1^ ^,^ ^2^ In Brazil, data from 2019 show 2,529 new cases of VL, with 1.2 cases per 100,000 inhabitants and a mortality rate of 9%. For CL, 15,484 new cases were confirmed (7.37 cases per 100,000 inhabitants), of which 67.1% had clinical cure 1.9% abandoned treatment and 19 deaths.^3^ There are at least eight species of *Leishmania* that can infect humans and lead to the development of parasitosis in Brazil: *L. (V.) braziliensis*, *L. guyanensis*, *L. (L.) amazonensis*, *L. (L.) infantum* (syn. chagasi) *L. (V.) lainsoni*, *L (V.) naiffi*, *L. (V.) shawi* and *L. (V.) lindenbergi*.^4^ Considering that Leishmaniasis is found on every continent except Australia, Pacific Islands and Antarctica, and there are about 90 countries classified as endemic, WHO estimates approximately 1.2 million new cases of cutaneous leishmaniasis per year.^2^ For visceral leishmaniasis, it is estimated that the new incidences are currently below 100,000 per year.^2^

The enzyme glutamine synthetase (GS) is essential in nitrogen metabolism, being responsible for the catalysis of glutamine from ATP, glutamate, and ammonia; this process occurs in two stages, starting with the activation of an intermediate gamma-glutamyl phosphate (γ-G-P), followed by a nucleophilic attack of ammonia in this intermediate releasing phosphate and forming glutamine.^5^ ^,^ ^6^ ^,^ ^7^ ^,^ ^8^ It is found in all organisms, including *Leishmania* sp, presenting three types: GS-I, found in most prokaryotes, GS-II, found in eukaryotes, and GS-III, found in some prokaryotes.^9^ The GS types I and II are dodecamers formed by two hexameric rings maintained mainly by hydrophobic interactions. The GS type III is formed by two hexameric rings associate across opposite interfaces, each ring has flipped 180º with respect to its position in the other two types.^10^ ^,^ ^11^ Glutamine synthesis sequence of *L. braziliensis* (LbGS) is formed by two pentameric rings interacting, probably, by hydrophobic interactions due to the conservation (in relation to HsGS) of the sequence rich in prolines and lysines. Hydrogen bonds and salt bridges sustain the interaction of monomers, being interface weaker in LbGS than HsGS.^12^

## MATERIALS AND METHODS

In this work, the nucleotide sequence encoding (GenBank CAM36993.1) the putative LbGS was cloned into a pET28a plasmid vector. A 42.35 kDa protein was obtained by overexpressing LbGS in the *Escherichia coli* (DE3) NiCo strain with 1 mM IPTG at 30ºC for four hours. The recombinant protein was purified from the soluble fraction of cellular lysate using a HisTrap column in the Akta Purifier system (GE Healthcare) using buffer A (Sodium phosphate buffer pH 7.4 10 mM, 500 mM NaCl, 40 mM imidazole) to equilibrate the column and a linear gradient of buffer B (pH 7.4 10 mM sodium phosphate buffer, 500 mM NaCl, 1 M imidazole) for elution.

We performed crosslinking experiments (XL) using the purified protein as previously described.^13^ The protein was digested with trypsin in the proportion of 1/50 (E/S) for 20 hours and the enzymatic reaction and was stopped by adding trifluoroacetic (0.4% v/v final). Subsequently, the peptides were quantified using the fluorometric test - Qubit 4.0^®^ (Invitrogen) according to the manufacturer’s recommendations. Each sample was desalted and concentrated using Stage-Tips (STop and Go-Extraction TIPs) according to literature.^14^ The peptide mixture was suspended in 0.1% formic acid and analysed as follows. An Ultimate 3000 (Thermo Fisher^®^) coupled online with a Fusion Lumos Orbitrap mass spectrometer (Thermo Fisher^®^) was used for generating the mass spectra data. The peptide mixture was chromatographically separated on a column (15 cm in length with a 75 μm I.D.) packed in-house with ReproSil-Pur C18-AQ 3 μm resin (Dr Maisch GmbH HPLC) with a flow of 250 nL/min from 5% to 50% ACN in 0.1% formic acid in a 140 min gradient. The Fusion Lumos Orbitrap was set to the data-dependent acquisition (DDA) mode to automatically switch between full-scan MS and MS/MS acquisition with 60s dynamic exclusion. Survey scans (200-1500 m/z) were acquired in the Orbitrap system with a resolution of 120,000 at m/z 200. The most intense ions captured in a 2s cycle time were selected, excluding those unassigned and in a 1+ charge state, sequentially isolated and HCD (Higher-energy collisional dissociation) fragmented using a normalised collision energy of 30. The fragment ions were analysed with a resolution of 30,000 at 200 *m/z*. The general mass spectrometric conditions were as follows: 2.5 kV spray voltage, no sheath or auxiliary gas flow, heated capillary temperature of 250ºC, predictive automatic gain control (AGC) enabled, and an S-lens RF level of 40%. Mass spectrometer scan functions and nLC solvent gradients were controlled by the Xcalibur 4.1 data system (Thermo Fisher^®^). Protein identification was performed using Pattern Lab for proteomics V available at http://www.patternlabforproteomics.org and a database containing 8,084 sequences of *L. braziliensis* downloaded from Uniprot. Results were filtered as described in the software’s bioinformatics protocol^15^ and only the protein of interest was identified, thus achieving 0% FDR. XL identification was performed with the Spectrum Identification Machine for Cross-Linked Peptides (SIM-XL) software that is freely available at http://www.patternlabforproteomics.org/sim-xl.^16^ The LbGS sequence from *L. braziliensis* was downloaded on March 29th, 2021, from the NCBI. The search parameters considered: fully tryptic peptide candidates with masses between 600 and 4800 Da, 20 ppm for precursor and fragment mass. The modifications were carbamidomethylation of cysteine and oxidation of methionine as fixed and variable, respectively. The files are available in proteomics.fiocruz.br/LbGS (Supplementary data). The distance of 11.4 Å between cross-linked lysines identified using SIM-XL (scores limit of 1.5 for intralinks and 2.0 for interlink)^16^ was used as an input for I-TASSER.^17^ Structural analysis and visual inspection were conducted with EBI-PISA,^18^ Pymol (The PyMOL Molecular Graphics System, Version 2.0 Schrödinger), Wincoot,^19^ and ChimeraX.^20^

## RESULTS AND DISCUSSION

**Fig. 1:**
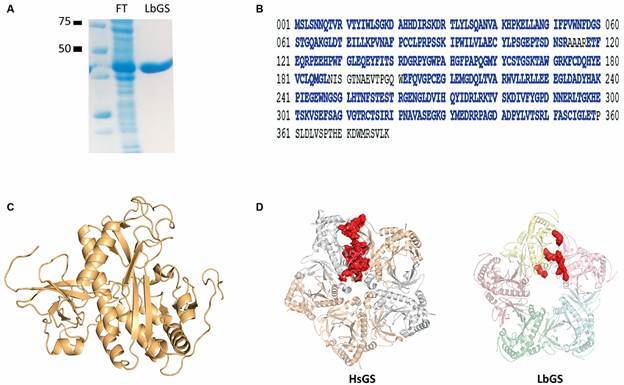
(A) purified recombinant glutamine synthetase (LbGS) (0.4 µg/µL). FT indicates the flow through of chromatography. (B) LbGS sequence with peptides identified by mass spectrometry in blue. (C) the tertiary model obtained with XLMS. (D) pentameric rings from HsGS (PDB ID 2OJW) and LbGS. In red the interacting interface residues.

The identity of purified protein (Fig. 1A) was confirmed by mass spectrometry (Fig. 1B). The experimental constraints obtained by XLMS are listed in Table I. The tertiary model (Fig. 1C) displayed a C-score value of 0.89 and a TM-score of 0.83 ± 0.08, which indicates good confidence and correct fold (TM-score > 0.5 suggests a correct fold).^17^ ^,^ ^21^ 37 out of 42 XL distances could be placed in the monomeric model, with acceptable distances between 11.4 Å and 35 Å (Table I).^22^

All GS are oligomers and eucaryotes GS type II are decamers composed of pentameric rings superimposed with monomers comprised of ~ 350 to 420 residues. GSs have ten active sites per oligomer placed in the interface of two interacting monomers.^5^ ^,^ ^23^ The 5 XL restraints that cannot be justified by a monomeric model should indicate the region of dimerization; these results corroborate that, like other GS, the dimerization occurs from the C-terminal region of one monomer and N-terminal of the other (Table I).

**TABLE I:** XL restrains obtained by XLMS and spatial distance between Ca in LbGS model

Residue 1 (Cα)	Residue 2 (Cα)	Distance (Å)
35/CA	41/CA	17,4
298/CA	308/CA	13,3
302/CA	282/CA	14,7
19/CA	298/CA	16,6
282/CA	298/CA	16,4
282/CA	317/CA	14
19/CA	317/CA	20,6
305/CA	298/CA	13,4
282/CA	302/CA	14,7
298/CA	278/CA	18,9
348/CA	298/CA	19,2
41/CA	298/CA	32,5
329/CA	317/CA	18,8
303/CA	278/CA	15,1
19/CA	329/CA	29,7
282/CA	303/CA	14,6
329/CA	298/CA	22,6
329/CA	303/CA	26,5
282/CA	28/CA	34,7
41/CA	317/CA	33,5
329/CA	348/CA	20,9
303/CA	329/CA	26,5
329/CA	308/CA	20,1
282/CA	329/CA	26,8
329/CA	41/CA	26,6
282/CA	308/CA	15,6
240/CA	259/CA	35.8
302/CA*	41/CA*	36*
44/CA*	302/CA*	37,2*
240/CA*	2/CA*	40,6*
19/CA*	326/CA*	35.1*
302/CA*	303/CA*	3.8*
303/CA*	298/CA*	9*
278/CA*	282/CA*	9.2*
163/CA*	167/CA*	9,3*
326/CA*	329/CA*	8.1*
281/CA*	282/CA*	3.9*
166/CA*	167/CA*	3,9*
17/CA*	19/CA*	6.6*
303/CA*	317/CA*	7.8*
329/CA*	329/CA*	0*

*: residues that cannot be explained by a monomeric model.

**Fig. 2:**
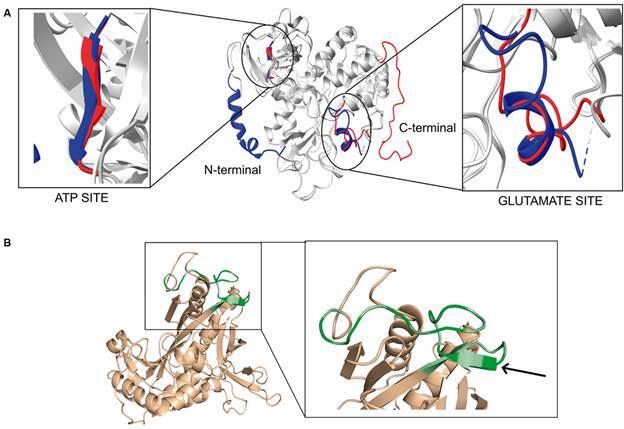
(A) superposing of glutamine synthetase (LbGS) and HsGS with differences highlighted. LbGS in red, HsGS in blue. (B) LbGS monomeric model with the region from L139 to P160 highlighted (green). Dark green indicates proline and glycine residues. Arrows indicate an β-sheet not conserved in the homolog HsGS.

The GS decamers present two main interaction interfaces: intra-ring forming the pentamers rings and tail-to-tail between superimposed pentameric rings. The human GS intra-ring interface is formed by the interaction of the N- and C-terminal of two subunits.^24^ Comparing LbGS and HsGS interfaces (Fig. 1D, Tables II-III), we observe a highly conserved C-terminal region but a divergent N-terminal (Fig. 2A). LbGS lacks an N-terminal a-helix which diminishes the size and strength of that interface (1537.4 Å^2^ and ΔG of -4.3 for LbGS and 2249.1 Å^2^ and ΔG of -15.4 for HsGS). The number of hydrogen bonds and salt bridges in HsGS are 37 and 16, respectively (Table II). In LbGS, the numbers are quite lower: seven hydrogen bonds and nine salt bridges (Table III). Although the interface of LbGS is less stable than HsGS, the following XLs might indicate that LbGS dimerization occurs *in solution*: K167-K166, S02-K240, D29-K28, C162-S163, K167-S166, D314-S317, D234-K240. Regarding the tail-to-tail interface, XL residues S163-K167 and S166-K167 can indicate the presence of this interface in our sample.

**TABLE II:** Intra-ring interface HsGS

Hydrogen bonds			
#	Structure 1	Distance (Å)	Structure 2
1	GLY 172	2,88	SER 06
2	ARG 181	2,97	MET 18
3	ARG 181	3,05	LEU 20
4	THR 193	3,07	THR 44
5	GLY 192	3,82	THR 46
6	TYR 180	2,81	THR 46
7	ARG 319	3,10	GLY 72
8	ARG 327	2,64	ASN 74
9	ARG 319	3,04	SER 75
10	ARG 327	2,78	ASP 76
11	ARG 327	3,23	ASP 76
12	ARG 324	3,19	ASP 76
13	ARG 324	3,16	ASP 76
14	ARG 319	3,12	ASP 76
15	ARG 181	3,18	ARG 90
16	ARG 327	3,67	TYR 104
17	ARG 173	2,90	GLU 230
18	GLY 166	2,76	GLU 230
19	ARG 173	2,67	GLU 230
20	GLY 148	2,66	SER 03
21	PRO 157	2,39	TYR 0
22	GLY 159	3,25	ARG 41
23	GLY 159	3,22	ARG 41
24	TYR 162	2,70	ARG 41
25	TYR 162	3,09	ASP 63
26	CYS 163	2,77	CYS 42
27	ALA 167	3,23	SER 03
28	ASP 174	2,56	LYS 11
29	GLU 177	2,97	ARG 90
30	GLU 177	2,87	ARG 90
31	TYR 180	3,39	THR 46
32	ILE 190	2,73	THR 46
33	ALA 191	2,85	THR 46
34	THR 193	3,10	THR 44
35	ASP 231	2,65	TYR 17
36	ALA 317	3,00	SER 75
37	ALA 317	3,07	SER 75
Salt bridges - HsGS			
#	Structure 1	Distance (Å)	Structure 2
1	ARG 319	3,89	ASP 63
2	ARG 324	3,65	ASP 76
3	ARG 324	3,19	ASP 76
4	ARG 324	3,16	ASP 76
5	ARG 319	3,12	ASP 76
6	ARG 324	3,56	ASP 76
7	ARG 173	3,67	GLU 230
8	ARG173	2,90	GLU 230
9	ARG 173	2,67	GLU 230
10	ARG 173	3,41	GLU 230
11	ASP 174	3,47	LYS 11
12	ASP 174	2,56	LYS 11
13	GLU 177	2,97	ARG 90
14	GLU 177	3,65	ARG 90
15	GLU 177	3,50	ARG 90
16	GLU 177	2,87	ARG 90

**TABLE III:** Intra-ring interface LbGS

Hydrogen bonds			
#	Structure 1	Distance (Å)	Structure 2
1	LYS 167	2,22	SER 140
2	THR 168	3,78	GLU 229
3	GLN 184	2,03	SER 02
4	THR 192	2,10	ASP 29
5	ARG 314	2,27	ASP 58
6	CYS 162	2,01	SER 27
7	SER 190	2,02	THR31
Salt bridges			
#	Structure 1	Distance (Å)	Structure 2
1	LYS 167	3,21	ASP 324
2	ARG 172	3,50	ASP 29
3	ARG 314	2,95	ASP 58
4	ARG 314	3,98	ASP 58
5	ARG 314	2,59	ASP 58
6	ARG 314	2,26	ASP 58
7	ARG 314	3,96	ASP 58
8	ARG 314	3,76	ASP 58
9	GLU 180	3,91	ARG 10

Studies have shown that the interaction of the pentameric HsGS model also depends on residues L139 to P160, which form a loop rich in proline and glycine, favoring hydrophobic interactions within pentamers.^24^ This loop (I138 to M159, LbGS numbering) is conserved in the LbGS structure and is also rich in prolines and glycines (Fig. 2B). However, the differences in protein sequence result in an β-sheet (R143, R144 and P145, LbGS numbering) not conserved in the homolog HsGS (Fig. 2B).

**Fig. 3:**
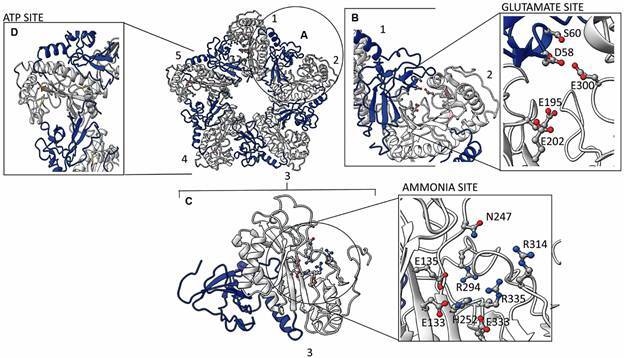
the active site of glutamine synthetase (LbGS). (A) in pentamer, each monomer is divided into two domains N-terminal (blue) and C-terminal (blue). (B) the glutamate site. (C) the ammonia site. (D) ATP site. Numbers references subunits of the pentameric ring.

The active site of GSs comprises three regions: one for glutamate, one for ATP and one for ammonia, with very conserved residues of two subunits of the pentameric ring.^23^ Each monomer is divided into two domains, each contributing with the active site of the adjacent monomer: N-terminal (smaller) is composed of a sheet formed by six antiparallel β-strands which two take parts of the active site; C-terminal (the larger), formed mainly by α-helix and six β-strands formed by most of the residues that make up the active site^9^ (Fig. 3A). In the model obtained by the crosslinking method, the active site residues E133, E135, N247, G248, H252, R294, R314, E333 and R335 (Glutamate site, LbGS numbering) (Fig. 3B), G186, S256 and R319 (ATP site, LbGS numbering) (Fig. 3C) are fully conserved together with the ammonia site, which involves three residues from the C-terminal region (E195, E202 and E300, LbGS numbering) and two residues from the N-terminal region (D58 and S60, LbGS numbering) from the adjacent subunit (Fig. 3D). The structural active sites differences found by superposing the GsHS and LbGS structures reside in: (i) glutamate site from 287-303 (LbGS numbering, Fig. 2A), while R299 (PDB_ID 2OJW), which is the terminal part of a loop and R294 (LbGS) which is part of an α -helix; (ii) the ammonia site from 55-63, being D63 (PDB_ID 2OJW) parts of a β-sheet, and D58 (LbGS), part of a loop (Fig. 2A).

**Fig. 4:**
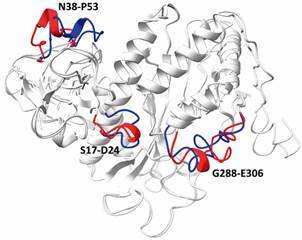
superposing of glutamine synthetase (LbGS) models generated with and without XL restrictions. The divergent regions S17-D24, N38-P53, G288-E306 from LbGS modeled with (blue) and without (red) restrictions are highlighted.

Finally, we also predicted a model using only the sequence of LbGS without XL restrictions. The model constructed by I-TASSER displayed good confidence scores (C-score value of -0.12, TM-score of 0.70 ± 0.12). When both LbGS models (modeled with and without XL restrictions) are superimposed, they present an RMSD of 0.671Å and the following regions would be modeled inappropriately in the absence of the restraints obtained from experimental XL: S17-D24, N38-P53, G288-E306 (Fig. 4). Thus, it is relevant to say that spatial restrictions give modeling a sense of the real conformation of the enzyme *in vitro*, differently from the model generated only based on homology allowing us to evaluate their *in-solution* conformation, in addition to the comparison with already known 3D counterparts structures available in the PDB. The technique of drug design aims both small therapeutic molecules targeting protein as itself as drug (biotherapeutics). Knowing proteins conformation *in vitro* and the ligand binding sites are the heart of structure-based drug development. Currently, this strategy depends not only on structural information, but also on dynamics, kinetics, and enzyme-substrate interaction data, that together provide the dynamic information on protein’s *in vitro* conformation and flexibility and are possible due to the computational advances that emerged over years.^25^ ^,^ ^26^ Some studies have used GS enzymes as a therapeutic target to treat diseases such as cancer, malaria, and leishmaniases.^23^ ^,^ ^24^ ^,^ ^27^ ^,^ ^28^

The LbGS lacks structural and functional studies being the studies with GS from *L. donovani* (LdGS) the closest to LbGS. Kumar et al performed biochemical studies that demonstrate the enzyme’s dependence on divalent metals for its optimal activity and optimum pHs from 7 to 9, similar to HsGS.^29^ Also, the provide a structural comparison of LdGS and HsGS describing relevant non-conserved residues for substrate recognition (E7, L132, S190, S249 and V205, LdGS numbering) and the importance of the electropositive potential in the active sites.^30^ These differences allowed them to find specific LdGS inhibitors, that might act in LbGS as we observed that the residues are conserved in LbGS. The potential of GS from *Leishmania* sp as therapeutic target was also evidenced by knock-out experiments indicating the dependence of parasite proliferation and infectivity on external supply of glutamine.^31^

Herein, we provide LbGS structural investigation identifying the active site, important interfaces, and unique structural features from LbGS. All these information allow investigation for new drugs.
