# Supplementary material for: Cross-linking mass spectrometry reveals structural insights of the glutamine synthetase from Leishmania braziliensis
Source: Mem Inst Oswaldo Cruz. 2022 Jan 10;116:e210209. doi: 10.1590/0074-02760210209 (PMC8752055; doi:10.1590/0074-02760210209)
Supplement: Supplementary file 1 [file 1678-8060-mioc-116-e210209-s.pdf]

The files are available in http://proteomics.fiocruz.br/lbgs_manuscript/

1. The annotated spectra are in the LbGS.sepr2 file; to view them, download the PLV (http://www.patternlabforproteomics.org/). Click on load to open the file, click on the protein, the table below will show the peptides, and then double click on the peptide to view the spectrum.

2. The annotated spectra from XL in the LbGS.simxlr file; to view them, download the SIM-XL (http://www.patternlabforproteomics.org/sim-xl). Right-click on the red line, pop-up window open, double-click in a row to view the spectrum.

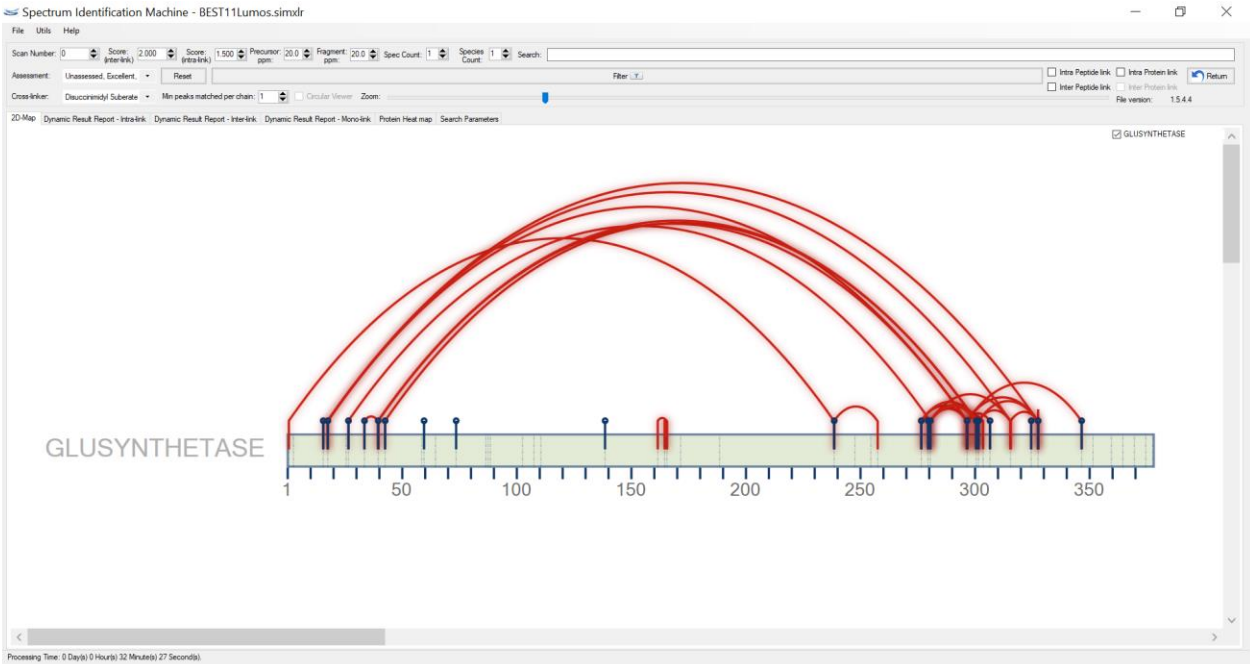

SUPPLEMENTARY DATA

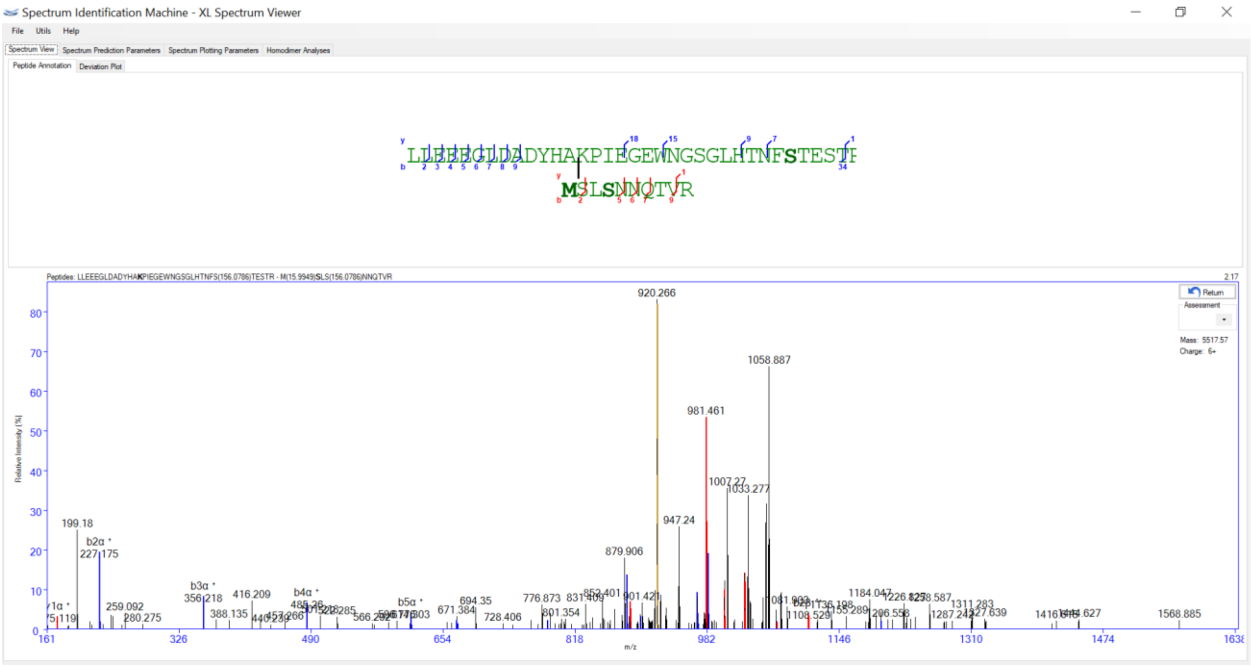
Spectrum Identification Machine - XL Spectrum Viewer
File Utils Help
Spectrum View Spectrum Prediction Parameters Spectrum Plotting Parameters Homodimer Analyses
Peptide Annotation Deviation Plot
Peptides: LLEEEGLDADYHAKPIEGEWNGSGLHTNFS(156.0786)TESTR - M(15.9949)SLS(156.0786)NNQTVR
Relative Intensity (%)
m/z
Return
Assessment
Mass: 5517.57
Charge: 6+

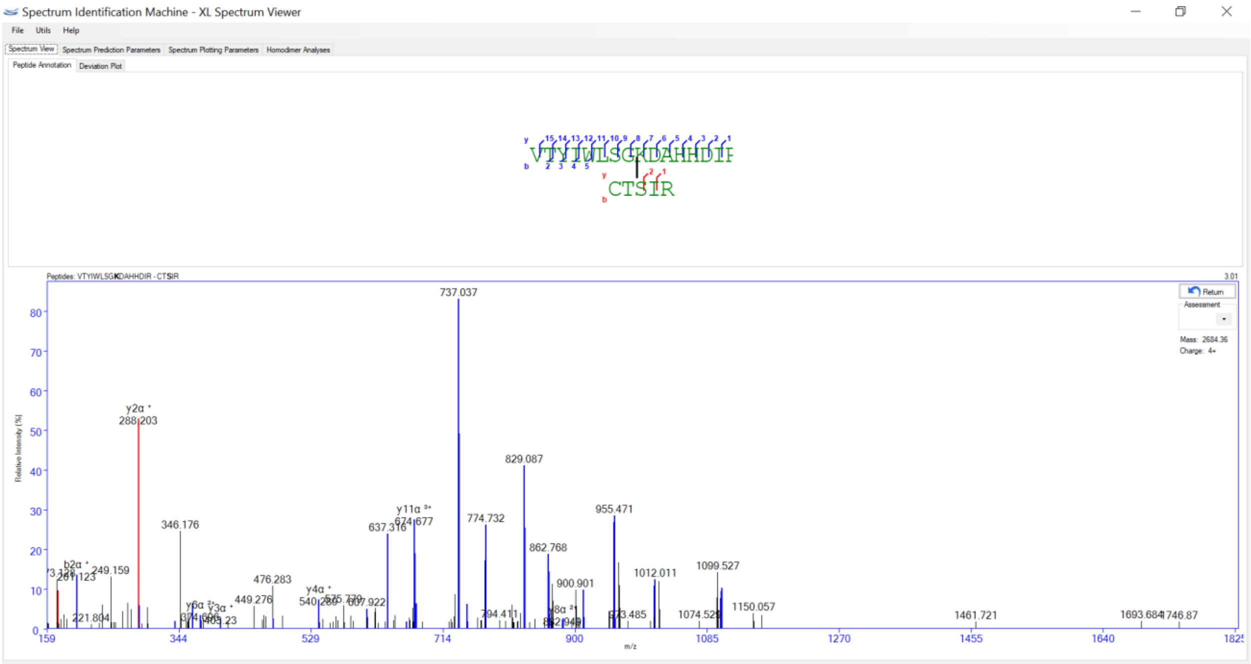
Spectrum Identification Machine - XL Spectrum Viewer
File Utils Help
Spectrum View Spectrum Prediction Parameters Spectrum Plotting Parameters Homodimer Analyses
Peptide Annotation Deviation Plot
Peptides: VTYIWLSGKDAHHDIR - CTSIR
Relative Intensity (%)
m/z
Return
Assessment
Mass: 2684.36
Charge: 4+

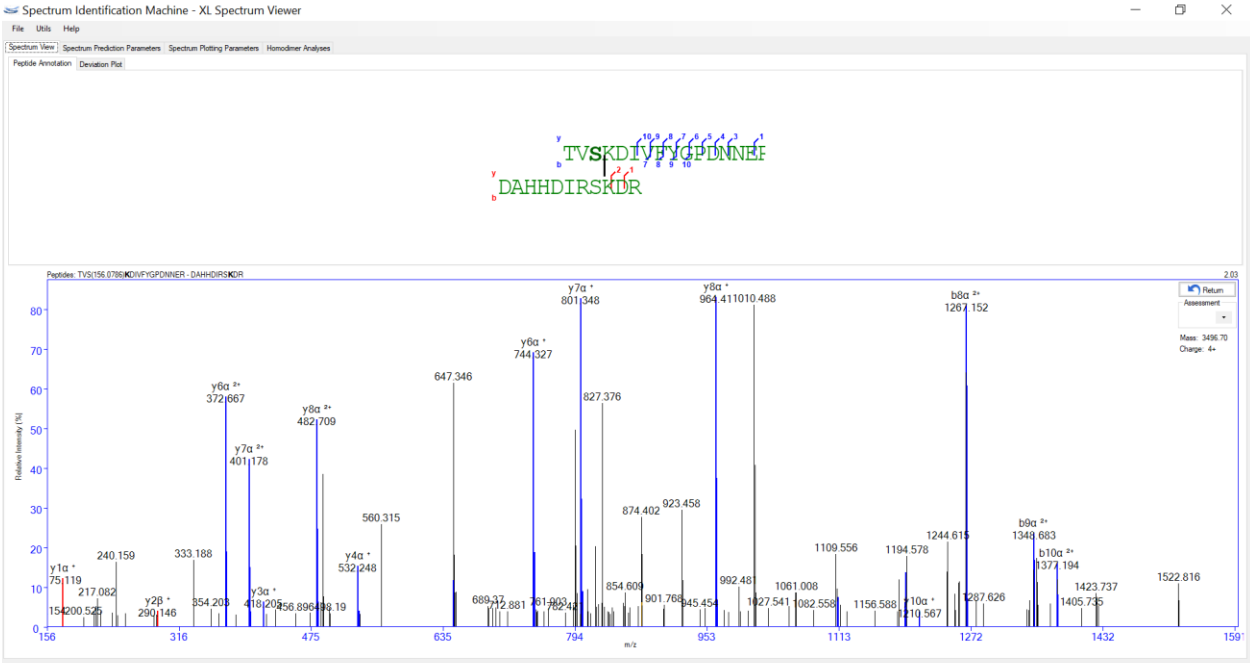
Spectrum Identification Machine - XL Spectrum Viewer
File Utils Help
Spectrum View Spectrum Prediction Parameters Spectrum Plotting Parameters Homodimer Analyses
Peptide Annotation Deviation Plot
TVSKDIVFYGPDNNER
DAHHDIRSKDR
Peptides: TVS(156.0786)KDIVFYGPDNNER - DAHHDIRSKDR
y7α + 801.348
y8α + 964.411
1010.488
b8α 2+ 1267.152
y6α + 744.327
647.346
y6α 2+ 372.667
827.376
y8α 2+ 482.709
y7α 2+ 401.178
874.402
923.458
560.315
1109.556
1194.578
1244.615
b9α 2+ 1348.683
b10α 2+ 1377.194
1522.816
1423.737
1405.735
240.159
333.188
y1α + 75.119
y4α + 532.248
854.609
992.481
1061.008
217.082
y2β + 290.146
y3α + 418.205
Relative Intensity (%)
m/z
Return
Assessment
Mass: 3496.70
Charge: 4+

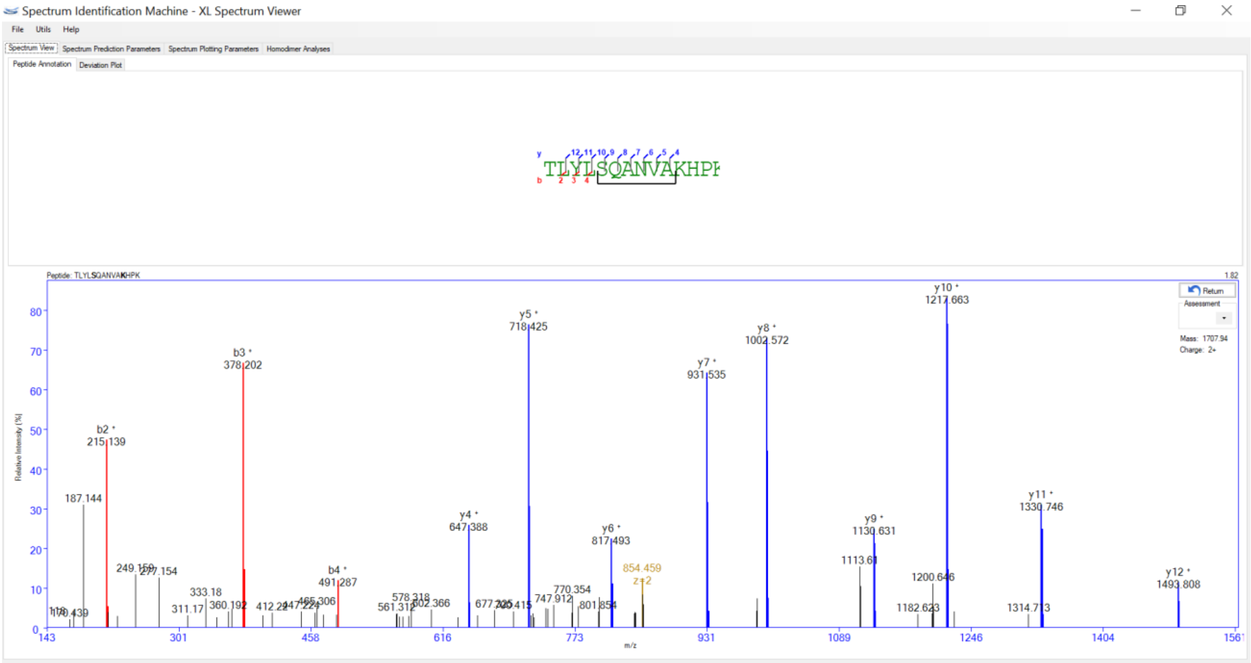
Spectrum Identification Machine - XL Spectrum Viewer
File Utils Help
Spectrum View Spectrum Prediction Parameters Spectrum Plotting Parameters Homodimer Analyses
Peptide Annotation Deviation Plot
TLYLSQANVAKHPK
Peptide: TLYLSQANVAKHPK
y10 + 1217.663
y5 + 718.425
y8 + 1002.572
b3 + 378.202
y7 + 931.535
b2 + 215.139
187.144
y11 + 1330.746
y4 + 647.388
y9 + 1130.631
y6 + 817.493
1113.61
249.159
277.154
b4 + 491.287
854.459
z=2
1200.646
y12 + 1493.808
770.354
747.912
333.18
1182.623
1314.713
Relative Intensity (%)
m/z
Return
Assessment
Mass: 1707.94
Charge: 2+

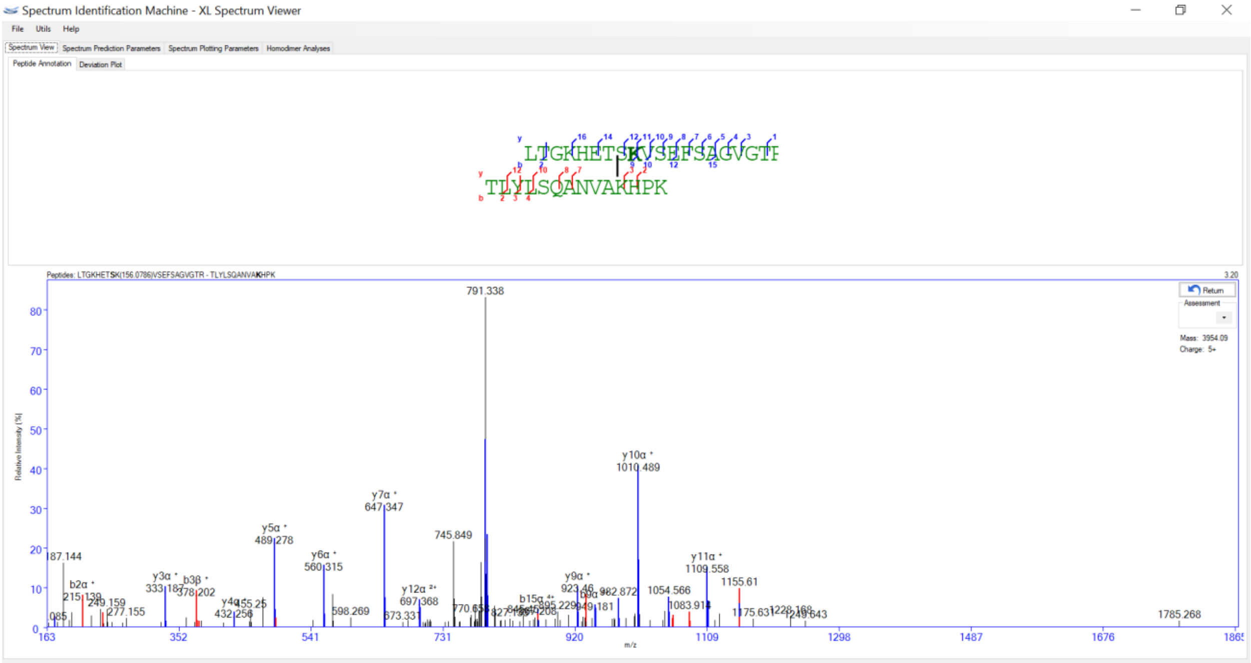
Spectrum Identification Machine - XL Spectrum Viewer
File Utils Help
Spectrum View
Spectrum Prediction Parameters
Spectrum Plotting Parameters
Homodimer Analyses
Peptide Annotation
Deviation Plot
LTGKHETSKVSEFSAGVGTR
TLYLSQANVAKHPK
Peptides: LTGKHETSK(156.0786)VSEFSAGVGTR - TLYLSQANVAKHPK
791.338
y10α⁺ 1010.489
y7α⁺ 647.347
745.849
y5α⁺ 489.278
y6α⁺ 560.315
y11α⁺ 1109.558
1155.61
187.144
1785.268
Relative Intensity (%)
m/z
Return
Assessment
Mass: 3954.09
Charge: 5+

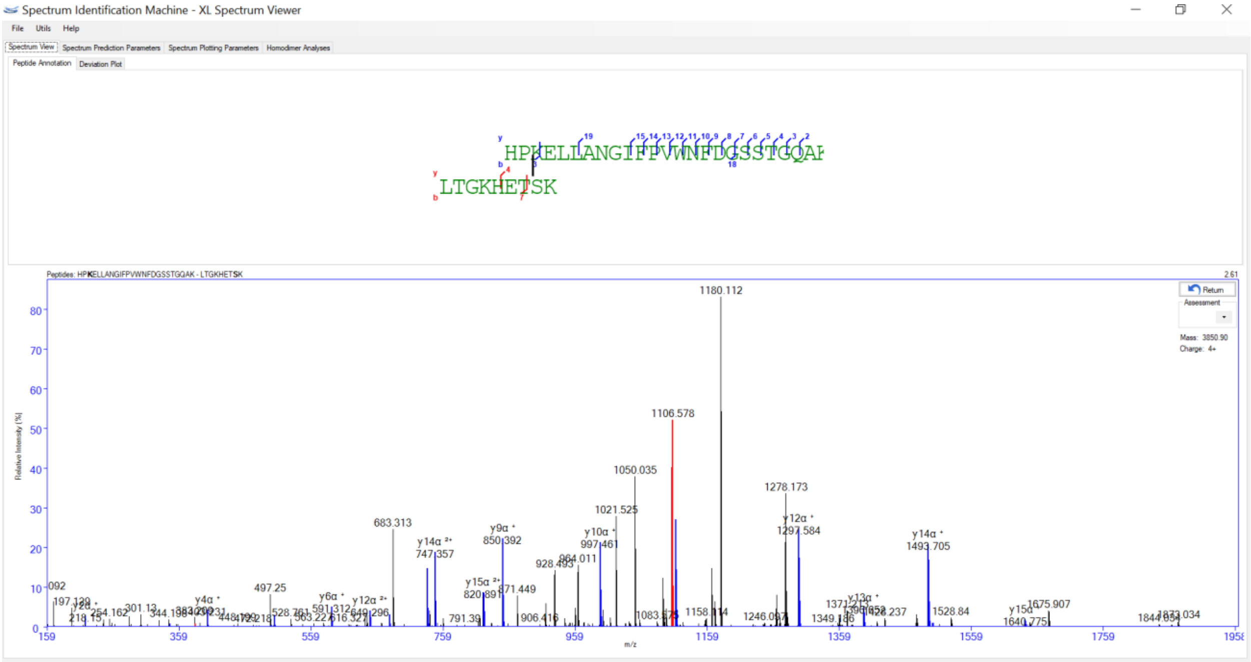
Spectrum Identification Machine - XL Spectrum Viewer
File Utils Help
Spectrum View
Spectrum Prediction Parameters
Spectrum Plotting Parameters
Homodimer Analyses
Peptide Annotation
Deviation Plot
HPKELLANGIFPVWNFDGSSTGQAK
LTGKHETSK
Peptides: HPKELLANGIFPVWNFDGSSTGQAK - LTGKHETSK
1180.112
1106.578
1050.035
1278.173
1021.525
683.313
y12α⁺ 1297.584
y14α⁺ 1493.705
y9α⁺ 850.392
y10α⁺ 997.461
1675.907
Relative Intensity (%)
m/z
Return
Assessment
Mass: 3850.90
Charge: 4+

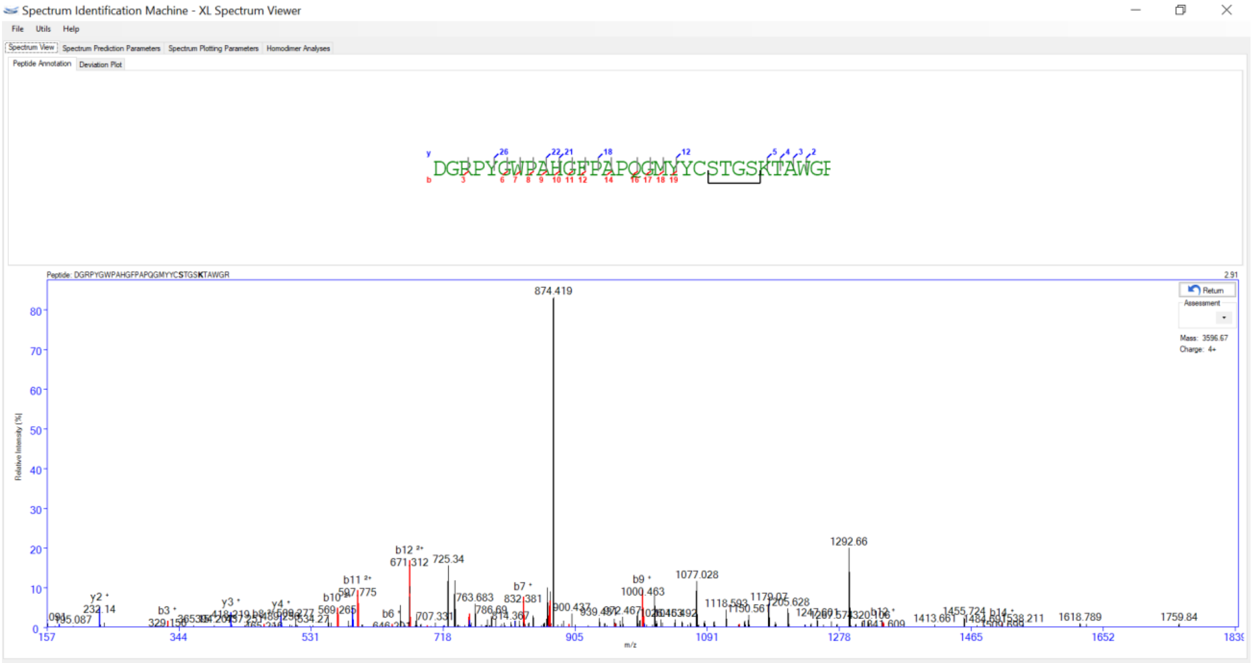
Spectrum Identification Machine - XL Spectrum Viewer
File Utils Help
Spectrum View Spectrum Prediction Parameters Spectrum Plotting Parameters Homodimer Analyses
Peptide Annotation Deviation Plot
Peptide: DGRPYGWPAHGFPAPQGMYYCSTGSKTAWGR
Relative Intensity (%)
m/z
874.419
1292.66
b12 2+ 671.312
725.34
1077.028
b9 + 1000.463
b11 2+ 597.775
b7 + 832.381
y2 + 232.14
b3 + 329.156
1618.789
1759.84
Mass: 3596.67
Charge: 4+
Return
Assessment

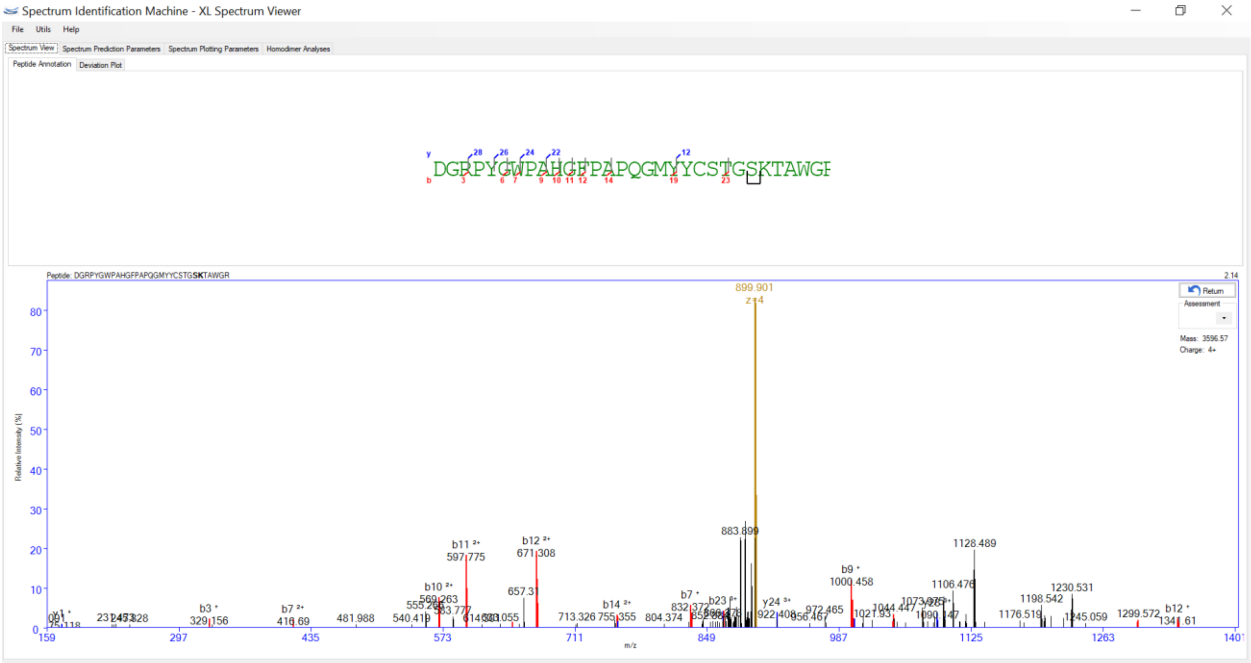
Spectrum Identification Machine - XL Spectrum Viewer
File Utils Help
Spectrum View Spectrum Prediction Parameters Spectrum Plotting Parameters Homodimer Analyses
Peptide Annotation Deviation Plot
Peptide: DGRPYGWPAHGFPAPQGMYYCSTGSKTAWGR
Relative Intensity (%)
m/z
899.901
z 4
883.899
1128.489
b11 2+ 597.775
b12 2+ 671.308
b9 + 1000.458
1106.476
1230.531
1198.542
b3 + 329.156
b7 2+ 416.69
481.988
Mass: 3596.57
Charge: 4+
Return
Assessment

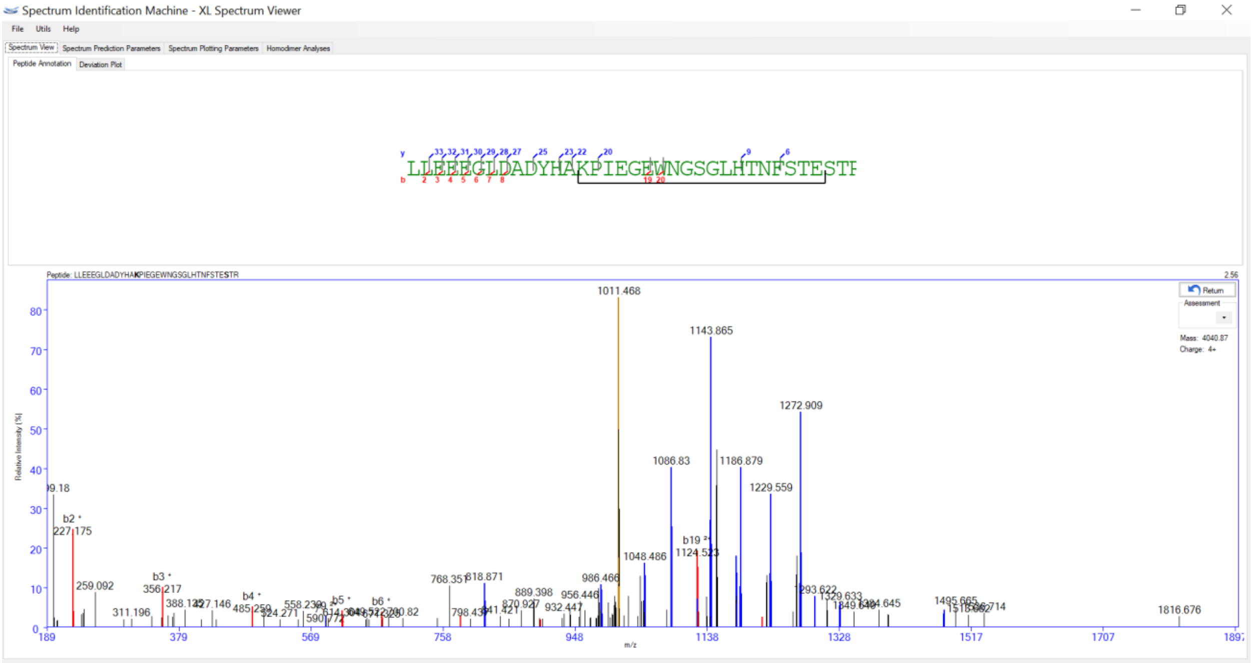
Spectrum Identification Machine - XL Spectrum Viewer
File
Utils
Help
Spectrum View
Spectrum Prediction Parameters
Spectrum Plotting Parameters
Homodimer Analyses
Peptide Annotation
Deviation Plot
Peptide: LLEEEGLDADYHAKPIEGEWNGSGLHTNFSTESTR
1011.468
1143.865
1272.909
1086.83
1186.879
1229.559
1048.486
1124.523
b19
b2
227.175
259.092
311.196
b3
356.217
b4
b5
b6
768.357
818.871
889.398
986.466
956.446
932.447
1816.676
Return
Assessment
Mass: 4040.87
Charge: 4+
Relative Intensity (%)
m/z
189
379
569
758
948
1138
1328
1517
1707

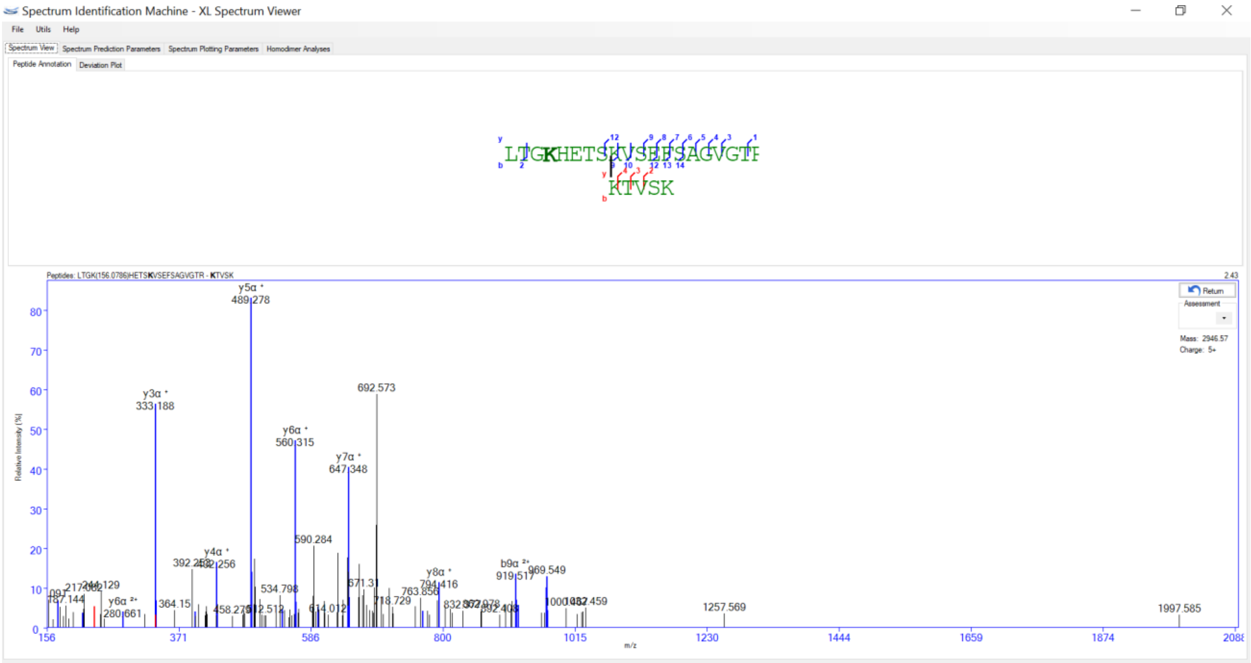
Spectrum Identification Machine - XL Spectrum Viewer
File
Utils
Help
Spectrum View
Spectrum Prediction Parameters
Spectrum Plotting Parameters
Homodimer Analyses
Peptide Annotation
Deviation Plot
Peptides: LTGK(156.0786)HETSKVSEFSAGVGTR - KTVSK
KTVSK
y5α
489.278
y3α
333.188
692.573
y6α
560.315
y7α
647.348
590.284
y4α
y8α
794.416
b9α
919.517
969.549
1257.569
1997.585
Return
Assessment
Mass: 2946.57
Charge: 5+
Relative Intensity (%)
m/z
156
371
586
800
1015
1230
1444
1659
1874

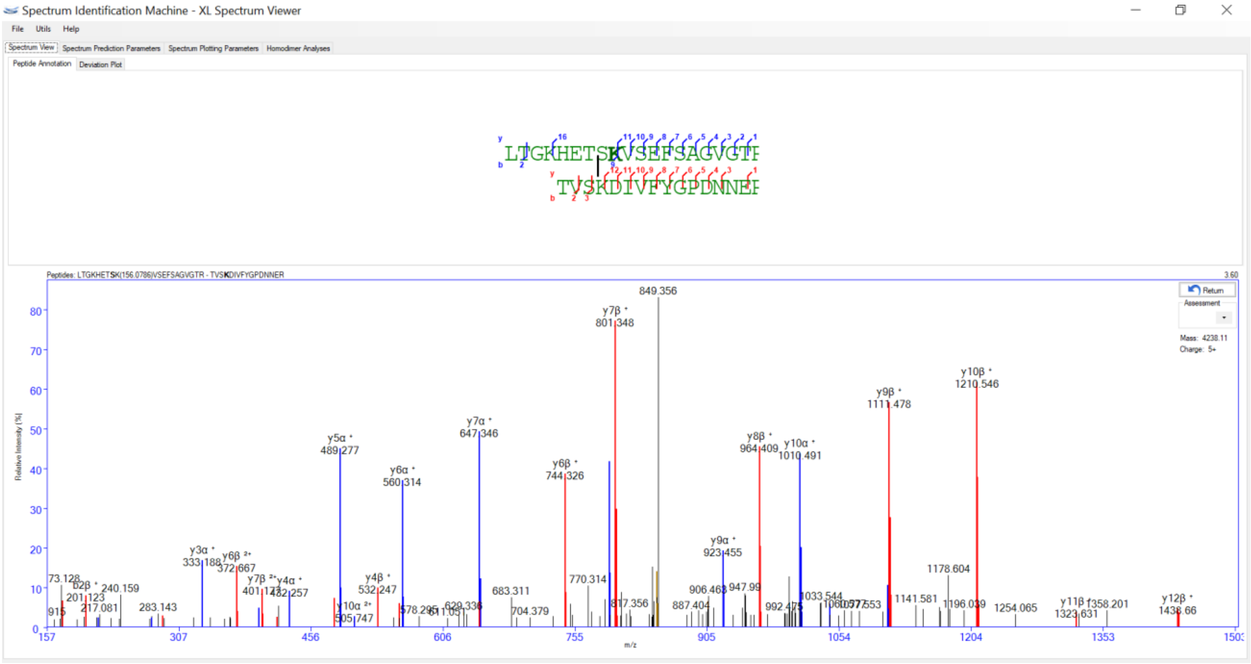
Spectrum Identification Machine - XL Spectrum Viewer
File Utils Help
Spectrum View Spectrum Prediction Parameters Spectrum Plotting Parameters Homodimer Analyses
Peptide Annotation Deviation Plot
Peptides: LTGKHETSK(156.0786)VSEFSAGVGTR - TVSKDIVFYGPDNNER
Relative Intensity (%)
m/z
849.356
y7β 801.348
y10β 1210.546
y9β 1111.478
y7α 647.346
y5α 489.277
y6α 560.314
y6β 744.326
y8β 964.409
y10α 1010.491
y9α 923.455
y3α 333.188
1178.604
Return
Assessment
Mass: 4238.11
Charge: 5+

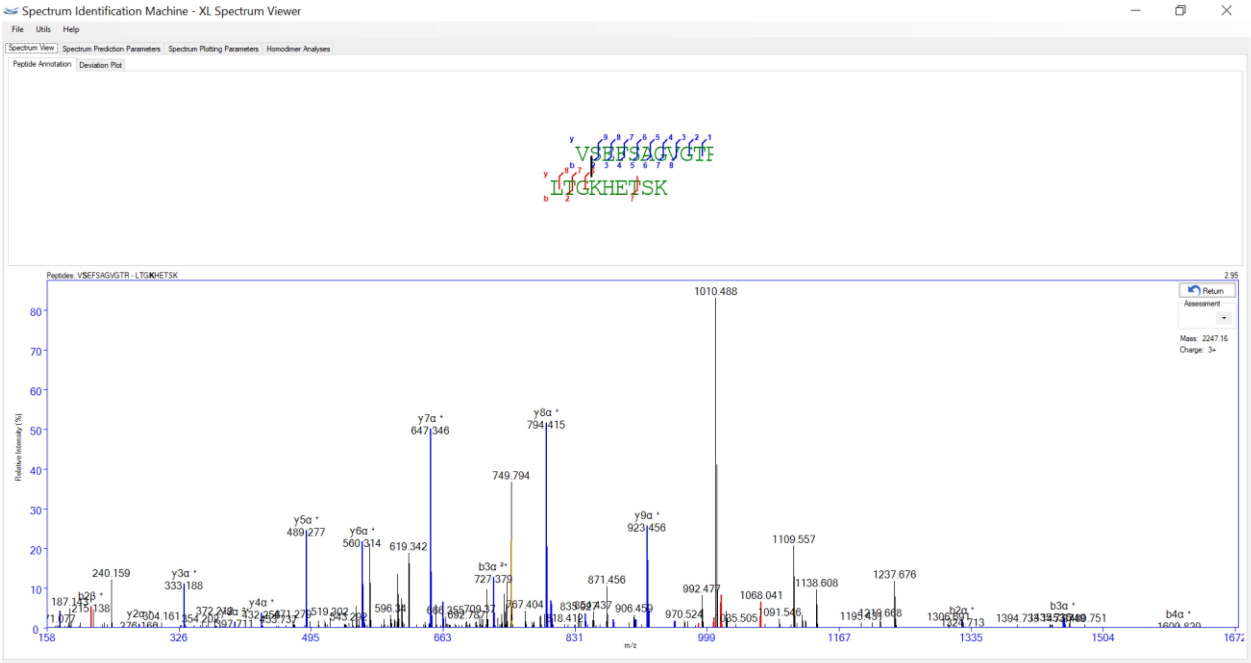
Spectrum Identification Machine - XL Spectrum Viewer
File Utils Help
Spectrum View Spectrum Prediction Parameters Spectrum Plotting Parameters Homodimer Analyses
Peptide Annotation Deviation Plot
Peptides: VSEFSAGVGTR - LTGKHETSK
Relative Intensity (%)
m/z
1010.488
y7α 647.346
y8α 794.415
749.794
y9α 923.456
y5α 489.277
y6α 560.314
619.342
1109.557
240.159
y3α 333.188
1237.676
1138.608
Return
Assessment
Mass: 2247.16
Charge: 3+

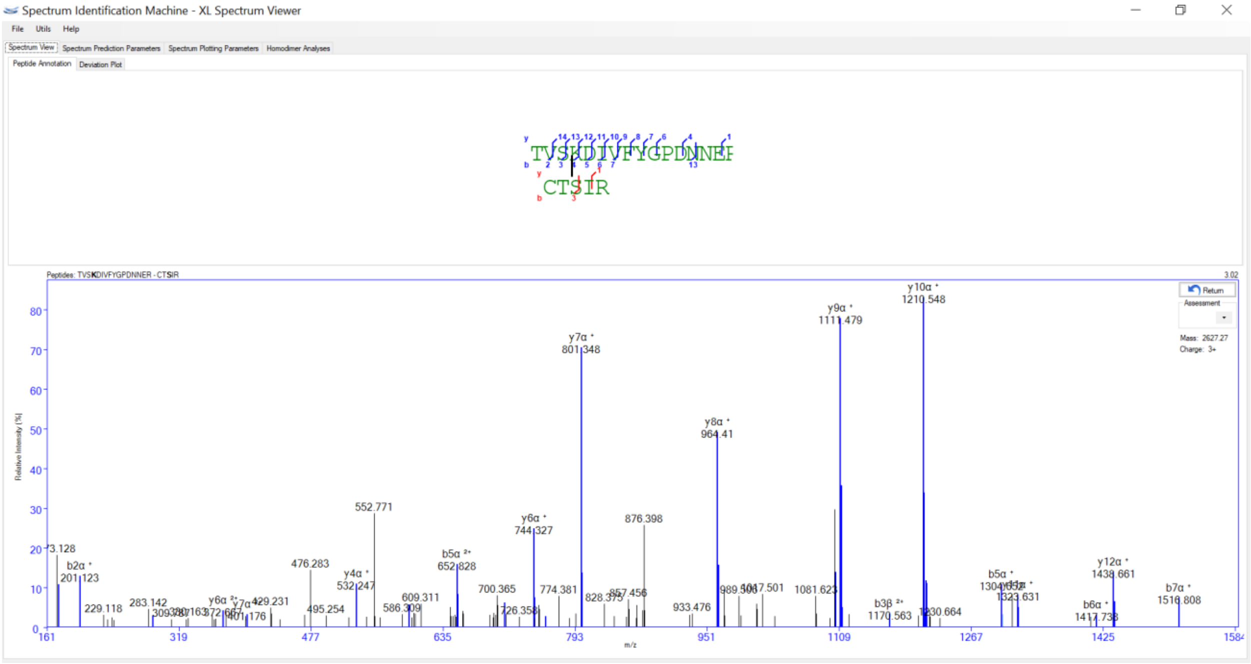
Spectrum Identification Machine - XL Spectrum Viewer
File Utils Help
Spectrum View Spectrum Prediction Parameters Spectrum Plotting Parameters Homodimer Analyses
Peptide Annotation Deviation Plot
TVSKDIVFYGPDNNEF
CTSIR
Peptides: TVSKDIVFYGPDNNER - CTSIR
y10α⁺ 1210.548
y9α⁺ 1111.479
y7α⁺ 801.348
y8α⁺ 964.41
552.771
876.398
y6α⁺ 744.327
b5α²⁺ 652.828
476.283
y12α⁺ 1438.661
b7α⁺ 1516.808
Mass: 2627.27
Charge: 3+
Relative Intensity (%)
m/z

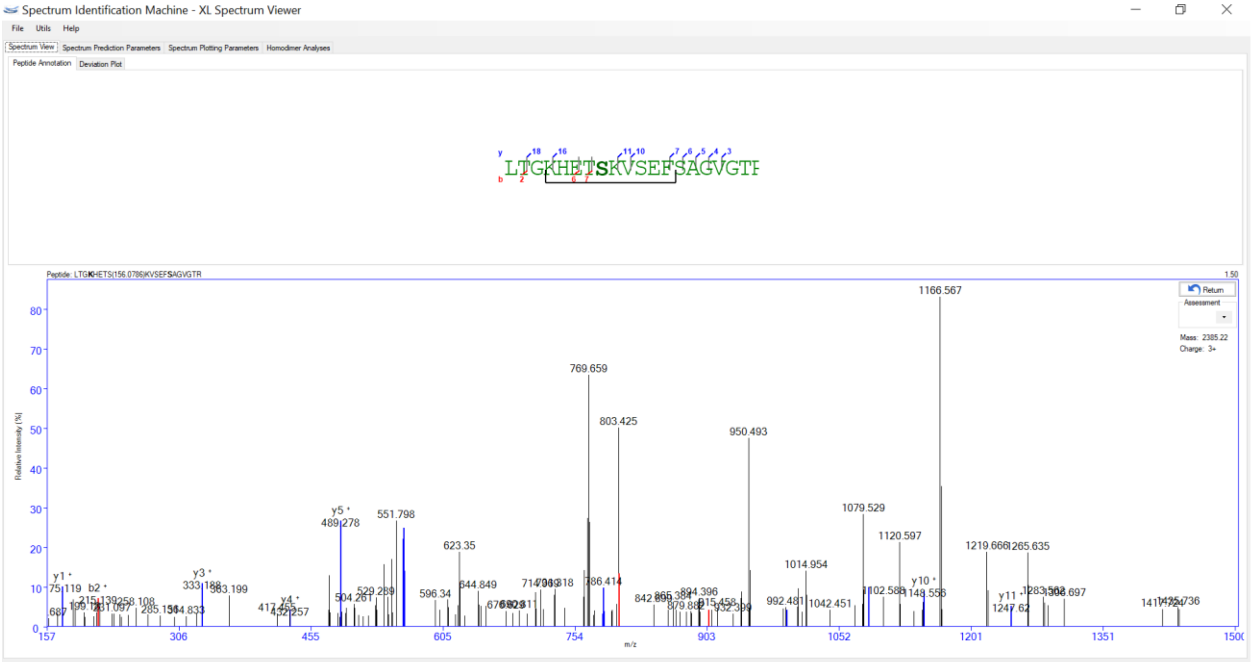
Spectrum Identification Machine - XL Spectrum Viewer
File Utils Help
Spectrum View Spectrum Prediction Parameters Spectrum Plotting Parameters Homodimer Analyses
Peptide Annotation Deviation Plot
LTGKHETSKVSEFSAGVGTF
Peptide: LTGKHETS(156.0786)KVSEFSAGVGTR
1166.567
769.659
803.425
950.493
1079.529
1120.597
y5⁺ 489.278
551.798
623.35
1219.666
1265.635
1014.954
Mass: 2385.22
Charge: 3+
Relative Intensity (%)
m/z

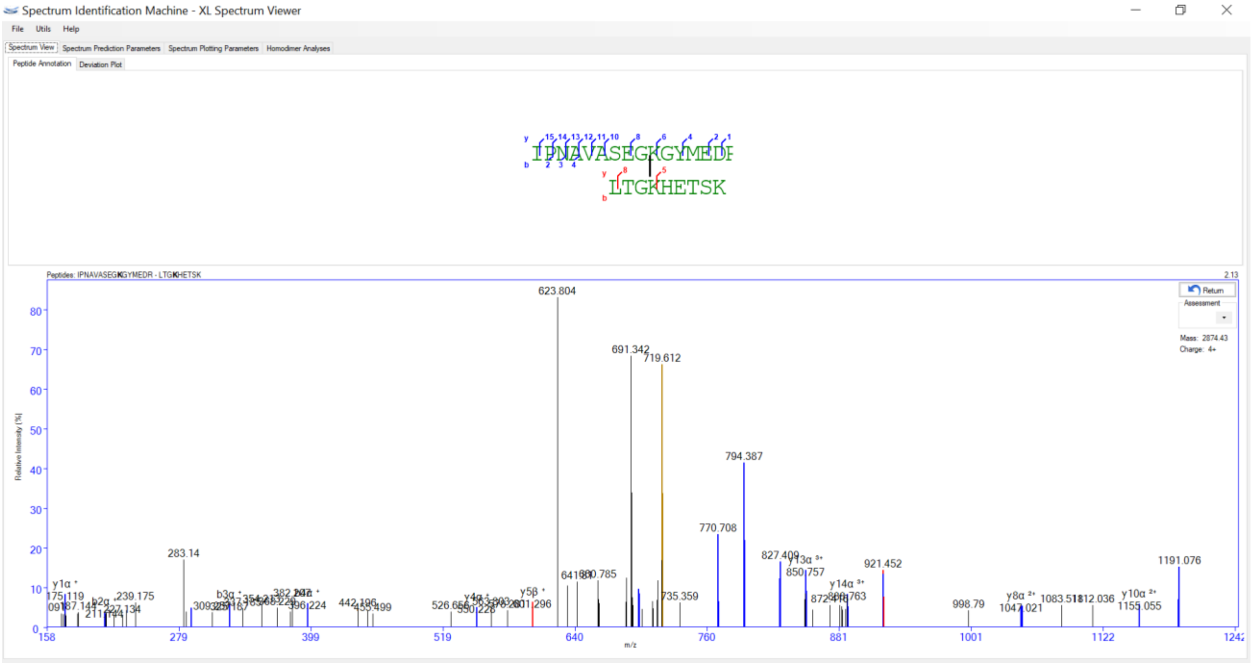
Spectrum Identification Machine - XL Spectrum Viewer
File Utils Help
Spectrum View Spectrum Prediction Parameters Spectrum Plotting Parameters Homodimer Analyses
Peptide Annotation Deviation Plot
Peptides: IPNAVASEGKGYMEDR - LTGKHETSK
Relative Intensity (%)
m/z
Mass: 2874.43
Charge: 4+
623.804
691.342
719.612
794.387
770.708
283.14
921.452
1191.076

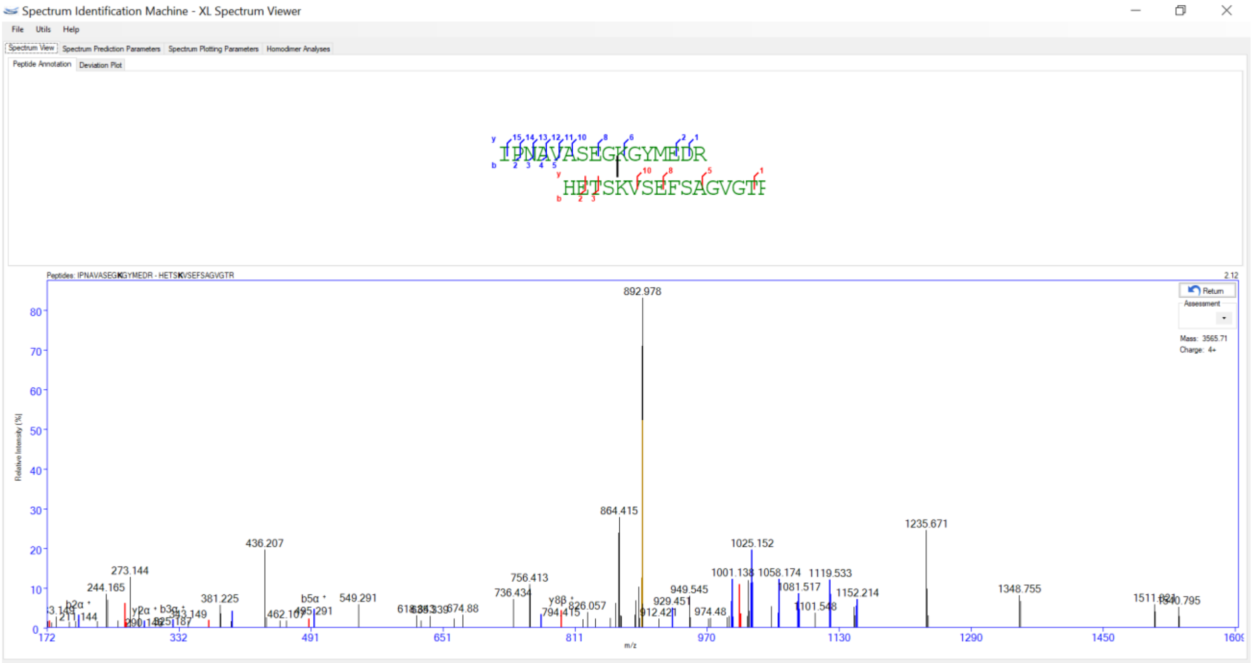
Spectrum Identification Machine - XL Spectrum Viewer
File Utils Help
Spectrum View Spectrum Prediction Parameters Spectrum Plotting Parameters Homodimer Analyses
Peptide Annotation Deviation Plot
Peptides: IPNAVASEGKGYMEDR - HETSKVSEFSAGVGTR
Relative Intensity (%)
m/z
Mass: 3565.71
Charge: 4+
892.978
864.415
1235.671
436.207
1025.152
273.144

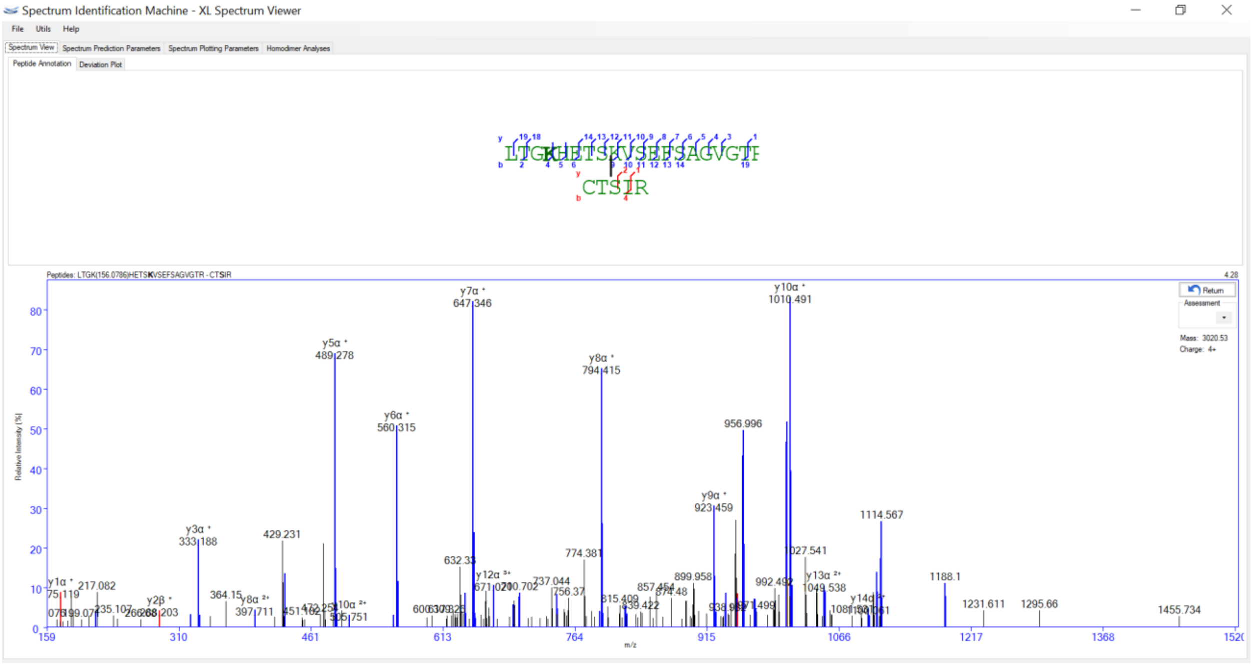
Spectrum Identification Machine - XL Spectrum Viewer
File Utils Help
Spectrum View Spectrum Prediction Parameters Spectrum Plotting Parameters Homodimer Analyses
Peptide Annotation Deviation Plot
CTSIR
Peptides: LTGK(156.0786)HETSKVSEFSAGVGTR - CTSIR
y7α + 647.346
y10α + 1010.491
y5α + 489.278
y8α + 794.415
y6α + 560.315
956.996
y9α + 923.459
1114.567
y3α + 333.188
429.231
774.381
1188.1
1231.611
1295.66
1455.734
Relative Intensity (%)
m/z
Mass: 3020.53
Charge: 4+
Return
Assessment

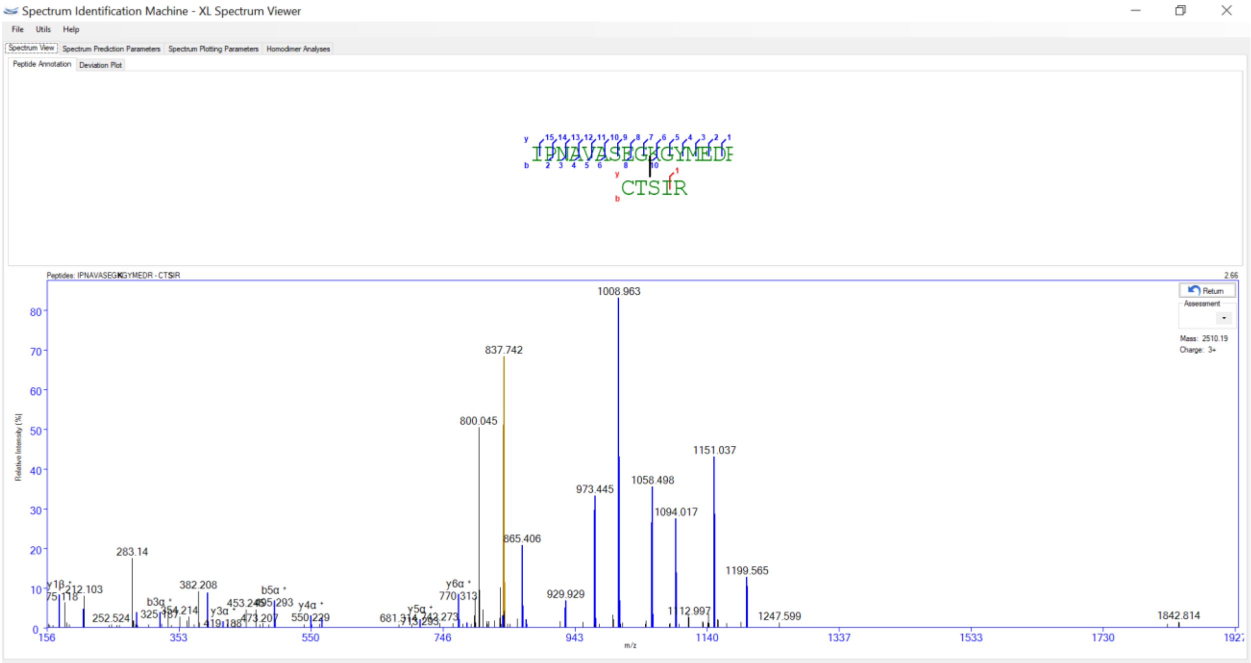
Spectrum Identification Machine - XL Spectrum Viewer
File Utils Help
Spectrum View Spectrum Prediction Parameters Spectrum Plotting Parameters Homodimer Analyses
Peptide Annotation Deviation Plot
CTSIR
Peptides: IPNAVASEGKGYMEDR - CTSIR
1008.963
837.742
800.045
1151.037
973.445
1058.498
1094.017
865.406
283.14
1199.565
382.208
929.929
1247.599
1842.814
Relative Intensity (%)
m/z
Mass: 2510.19
Charge: 3+
Return
Assessment

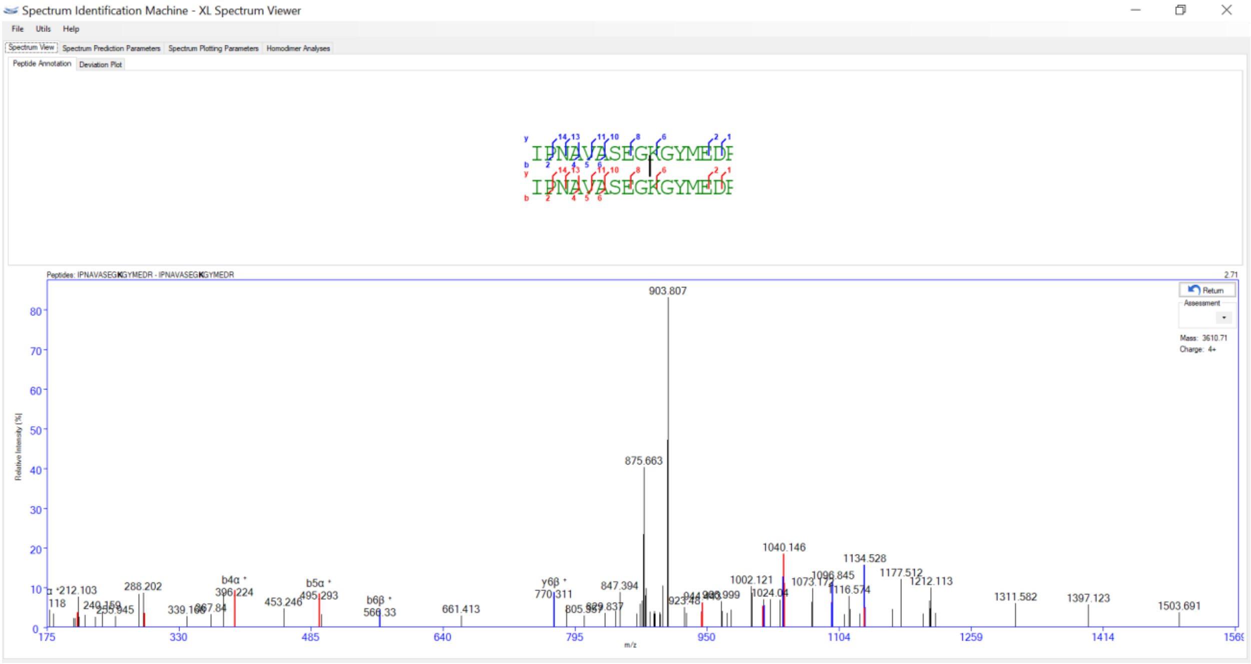
Spectrum Identification Machine - XL Spectrum Viewer
File Utils Help
Spectrum View
Spectrum Prediction Parameters
Spectrum Plotting Parameters
Homodimer Analyses
Peptide Annotation
Deviation Plot
Peptides: IPNAVASEGKGYMEDR - IPNAVASEGKGYMEDR
Relative Intensity [%]
m/z
Mass: 3610.71
Charge: 4+
903.807
875.663
1040.146
1134.528
1177.512
1212.113
1311.582
1397.123
1503.691
661.413
847.394
288.202
453.246

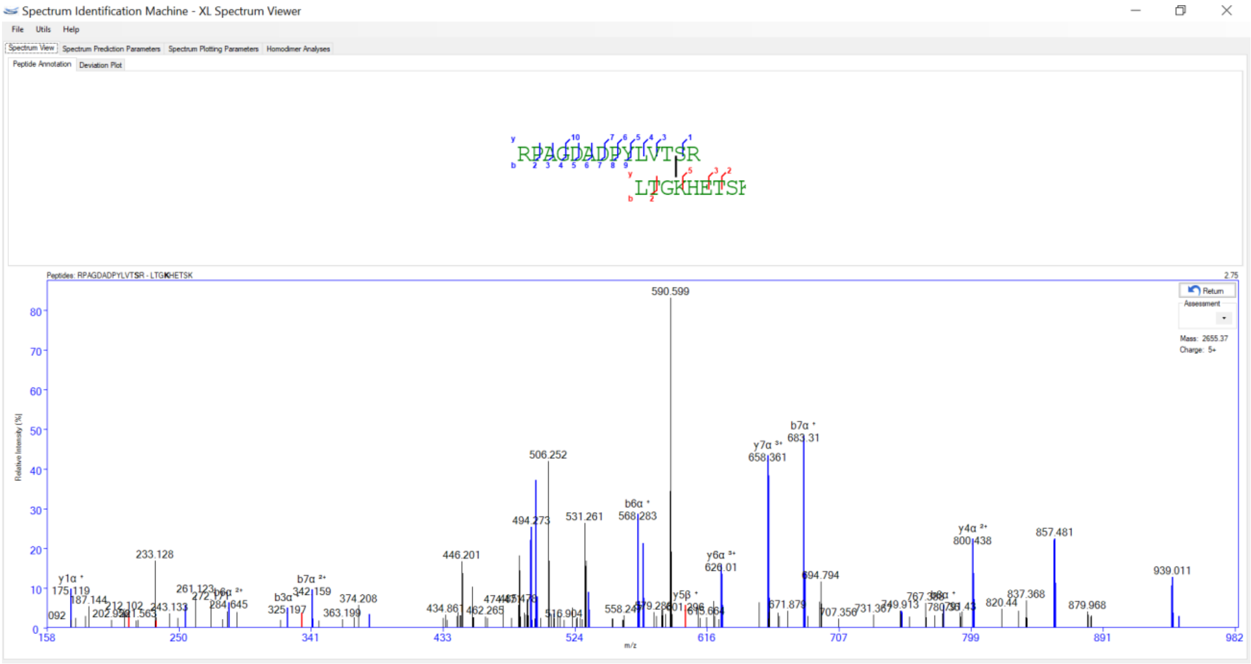
Spectrum Identification Machine - XL Spectrum Viewer
File Utils Help
Spectrum View
Spectrum Prediction Parameters
Spectrum Plotting Parameters
Homodimer Analyses
Peptide Annotation
Deviation Plot
Peptides: RPAGDADPYLVTSR - LTGKHETSK
Relative Intensity [%]
m/z
Mass: 2655.37
Charge: 5+
590.599
506.252
531.261
446.201
233.128
857.481
939.011
694.794
837.368
879.968
374.208
